# Influence of Deposition Time on Titanium Nitride (TiN) Thin Film Coating Synthesis Using Chemical Vapour Deposition

**DOI:** 10.3390/ma16134611

**Published:** 2023-06-26

**Authors:** Ranjan Kumar Ghadai, Kamaraj Logesh, Robert Čep, Jasgurpreet Singh Chohan, Kanak Kalita

**Affiliations:** 1 Department of Mechanical Engineering, Sikkim Manipal Institute of Technology, Sikkim Manipal University, Majhitar 737 136, India; ranjan.g@smit.smu.edu.in; 2 Department of Mechanical Engineering, Vel Tech Rangarajan Dr. Sagunthala R&D Institute of Science and Technology, Avadi 600 062, India; klogesh@veltech.edu.in; 3 Department of Machining, Assembly and Engineering Metrology, Faculty of Mechanical Engineering, VSB-Technical University of Ostrava, 708 00 Ostrava, Czech Republic; robert.cep@vsb.cz; 4 Department of Mechanical Engineering and University Centre for Research & Development, Chandigarh University, Mohali 140 413, India; jasgurpreet.me@cumail.in

**Keywords:** TiN, CVD, SEM, nanoindentation

## Abstract

Titanium nitride (TiN) thin film coatings were grown over silicon (p-type) substrate using the atmospheric pressure chemical vapour deposition (APCVD) technique. The synthesis process was carried out to evaluate the effect of deposition time on the physical and mechanical characteristics of TiN coating. Thin films grown over Si substrate were further characterised to evaluate the morphological properties, surface roughness and mechanical properties using a scanning electrode microscope (SEM), atomic force microscopy (AFM) and nanoindentation, respectively. EDS equipped with SEM showed the presence of Ti and N elements in considerable amounts. TiN morphology obtained from the SEM test showed small-sized particles on the surface along with cracks and pores. AFM results revealed that by increasing the deposition time, the surface roughness of the coating also increased. The nanomechanical properties such as nanohardness (H) and Young’s modulus (E), etc., evaluated using the nanoindentation technique showed that higher deposition time led to an increase in H and E. Overall, it was observed that deposition time plays a vital role in the TiN coating deposition using the CVD technique.

## 1. Introduction

Titanium nitride (TiN) thin film coating is a very well-known surface modification method that is extensively implemented in various industrial domains due to its unique properties. Basically, TiN is a very hard, wear-resistant material that is known for its excellent chemical stability, high melting point and low coefficient of friction [1]. These properties make it an ideal coating material for a range of applications, including cutting tools, medical implants, aerospace components and decorative coatings [2,3]. One of the most common applications of TiN coatings is in cutting tools. TiN coatings can significantly improve the performance and lifespan of cutting tools, such as drills, end mills and saw blades, by reducing wear and increasing hardness (H) [4]. This is achieved by growing a layer of TiN over the surface of the tool, which acts as a barrier against the abrasive forces of the material being cut. TiN-coated cutting tools are commonly used in the automotive, aerospace and manufacturing industries, where high precision and durability are critical. The use of TiN coatings in aerospace has helped to improve safety and reliability while reducing maintenance costs [5,6,7]. TiN coatings are also used in decorative applications, where their unique gold colour and high hardness make them an attractive option for jewellery and watch manufacturers. The coating can be applied to a variety of materials, including stainless steel, brass and titanium, to provide a durable and scratch-resistant finish. TiN-coated jewellery and watches are increasingly popular among consumers who are looking for high-quality, long-lasting products [8,9]. The performance of TiN coating intended for a desired application depends on various factors such as deposition condition, deposition technique used, deposition parameters, etc. [10]. There are various techniques available for the deposition of thin film such as physical vapour deposition (PVD), chemical vapour deposition (CVD), vacuum evaporation, molecular beam epitaxy, ion plating, etc. However, TiN can be synthesised using techniques such as physical vapour deposition (PVD), chemical vapour deposition (CVD) [11,12,13], etc. In PVD, TiN is synthesised by depositing thin films of TiN onto a substrate using a physical process, such as sputtering. In CVD, TiN is synthesised by reacting gases containing titanium and nitrogen ($N_{2}$) on a heated substrate. While both PVD and CVD techniques can produce high-quality TiN coatings, there are several advantages to using CVD for TiN synthesis. CVD can produce thicker coatings than PVD and the process can be easily scaled up for mass production [14]. CVD also offers greater control over the composition and properties of the TiN coatings, as well as the ability to coat complex geometries. Additionally, CVD can produce TiN coatings with excellent adhesion and uniformity, making them ideal for high-performance applications.

In this research article, the focus is on the synthesis and characterisation of TiN thin films deposited on silicon (Si) substrates. A CVD technique is employed to grow the thin films. The deposition time is varied in this study to analyse the effect on the film’s properties. After the synthesis, the samples are characterised using various analytical techniques to determine their structural, morphological and physical properties.

## 2. Literature Review

TiN thin films have been widely investigated due to their exceptional mechanical, electrical and optical properties [15]. Several studies have studied the synthesis and characterisation of TiN thin films using CVD and other deposition techniques. Sobell and George et al. [16] researched the properties of TiN and TiC coating grown using plasma-assisted CVD (PACVD). Krishna et al. [17] synthesised a multilayer thin film material consisting of carbon nanotubes (CNTs) and TiN and SiO_2_ coatings using radio frequency plasma-enhanced CVD (RF PECVD) and PVD. Ge et al. [18] investigated the correlation between the process parameters, microstructure and hardness of titanium nitride films deposited using CVD. Das et al. [19,20] investigated the influence of N_2_ flow rates on the properties of chemical vapour deposited TiN and titanium aluminium nitride (TiAlN) coatings. Valour et al. [21] presented the sol–gel method and rapid thermal nitridation for TiN thin film manufacturing.

Various studies have been conducted to study the mechanical properties of TiN and its derivatives, such as TiAlN and titanium carbon nitride (TiCN), when applied to cutting tools [22]. Aditharajan et al. [23] provided a comprehensive review of coating techniques with special emphasis on improvement of mechanical properties of cutting tools. Das et al. [24] studied the applicability of CVD for TiCN coatings for machine tools and characterised its morphological, structural and corrosion behaviour and mechanical properties. Subhedar et al. [25] investigated TiN-coated milling tools for aluminium milling, prepared using PVD, and found improved performance compared to uncoated tools.

Deposition temperature has been shown to significantly influence the properties of TiN thin films. Das et al. [26] deposited TiN thin films on Si substrates using thermal CVD at different temperatures and characterised their properties using various techniques. They found that TiN coating synthesised at 1150 °C had the maximum H, fracture toughness and E. In a similar study, the surface microstructures of TiN films were studied by Ma et al. [27]. They investigated the effect of substrate negative bias and N_2_ flow. During DC-reactive magnetron sputtering and PECVD deposition, they observed a higher H^3^/E^2^ ratio for TiN/Si3N4 films with higher H.

Additionally, TiN thin films have been employed in microheater applications. Jithin et al. [28] investigated the texture formation in TiN films grown using CVD.

The influence of deposition conditions on TiN and its derivatives’ properties has also been widely investigated. Das et al. [15] deposited TiN, TiAlN and TiAlSiN coatings over Si (100) substrates. They employed a constant temperature but different N_2_ flow rates in a CVD process. They observed that TiN coatings had a smoother surface compared to other coatings. They reported that increase in N2 increased the TiN/TiAlN particle size.

Several studies have also investigated the combination of TiN with other elements or compounds to enhance its properties. Guha et al. [29] deposited titanium silicon nitride thin films using various N_2_ flow rates using CVD. They found that increasing N_2_ flow rates increased surface roughness but improved the H and E of the films. In another study, Price et al. [30] investigated the properties of TiN coating prepared using the CVD process. The authors concluded that TiN coating grown using CVD showed excellent properties at higher deposition temperature. Su et al. [31] grew TiN films using CVD. Bull et al. [32] investigated the influence of Ti interlayers on the adhesion properties of TiN coating grown using the PACVD process. The authors observed improved adhesion with increased interlayer thickness of the PACVD-deposited TiN coating compared to the PVD-deposited coating. Jian et al. [33] reported the effect of variation in N content on the structural properties of TiN coating deposited using CVD. The results revealed an increase in lattice parameter with the increase in N content. Choi et al. [34] used indium tin oxide (ITO) and TiN thin films as interlayers to improve the adhesion between carbon nanowalls (CNWs) and substrates.

Atomic layer deposition (ALD) has also been utilised for depositing titanium-containing thin films. Zhang [35] provided an overview of the current technology of deposition of titanium-containing chemicals using ALD and their potential applications in various fields. In a similar vein, Robinson et al. [36] demonstrated the growth of continuous, stoichiometric SnTe thin films using a single-source CVD precursor and showed that they could selectively grow SnTe onto the TiN regions of SiO_2_/TiN-patterned substrates.

The corrosion resistance and tribological properties of TiN coatings have also been studied by various researchers. Grabarczyk et al. [37] investigated the influence of thermo-chemical treatment on the corrosion resistance and tribological properties of Ti-6Al-4V alloy. They found that oxidation resulted in a 200% higher H and improved corrosion resistance, while carburizing reduced the wear rate and the coefficient of friction. Moreover, Das et al. [15] conducted electrochemical tests and found that TiAlSiN coating exhibited higher corrosion resistance compared to other coatings.

In addition to TiN, researchers have explored the deposition of other thin film materials using CVD and related techniques. For instance, Jedrzejewska-Szczerska et al. [38] presented fibreoptic sensors based on nanolayers or thin films made from various materials, such as nanodiamond, zinc oxide, titanium dioxide and aluminium oxide, which were successfully applied in biosensing. Furthermore, Schade et al. [39,40] investigated the disintegration characteristics of silicon nitride in an artificial ocular setting, taking into account temperature and ion concentration variables. They discovered that the films gradually dissolved in saline solutions, and the application of a sputtered titanium oxide protective layer helped to decrease the dissolution rates.

The literature highlights the importance of various process parameters in determining the properties and performance of TiN thin films and their derivatives. Numerous studies have been conducted in evaluating the properties of TiN coating deposited by either varying the deposition temperature or the precursor gas flow rate. However, no literature related to property analysis of TiN coating deposited by varying the deposition tie has been published to the best of the authors’ knowledge. Moreover, the effect of deposition temperature and N_2_ flow rate on the synthesis, characterisation and application of these materials, with particular emphasis on their mechanical, electrical and optical properties, have been investigated frequently. By understanding the influence of deposition time on TiN thin film coating synthesis using CVD, researchers can better optimise the process parameters and develop coatings with improved performance for various applications, such as cutting tools, microheaters and protective overcoats. Table 1 represents the key findings in the literature related to TiN deposition.

## 3. Experimental Details

### 3.1. TiN Synthesis Details

TiN coatings were synthesised over p-type silicon (Si) substrate with dimensions 10 mm × 10 mm × 3 mm over the sighing surface using atmospheric pressure chemical vapour deposition (APCVD). The Si substrate, before being placed inside the CVD furnace, was washed thoroughly using the standard Radio Corporation of America (RCA) cleaning procedure [19]. All unwanted gases were thoroughly removed by keeping the furnace chamber pressure at 760 Torr. After successful extraction of unwanted gases from the heating chamber, the substrate was placed over an inverted ceramic crucible and kept inside the CVD chamber. The ceramic boat, consisting of TiO_2_ (99.99% pure) in powder form, approximately 20 gm, was kept 6 cm behind the substrate boat. For conducting the characterisation test, in each set of experiments, four Si substrates were used. Here, TiN synthesis was carried out by varying the deposition time from 30 min to 120 min. N_2_ gas was used as precursor gas for the deposition of TiN coating over the Si substrate. During all sets of experiments, the flow rate of N_2_ gas kept constant at 10 sccm (standard cubic centimetre). After the furnace was switched on, the base pressure and working pressure were set as t 0.75 mTorr and 500 m Torr, respectively. The deposition temperature for all sets of experiments was kept at 900 °C.

The process of TiN coating growth over the Si substrate carried out inside the CVD furnace is mentioned below:

1. Decomposition of TiO_2_ powder: at CVD furnace temperature of 900 °C, TiO_2_ (in powder form) decomposes to form titanium (Ti) and oxygen (O) atoms.
2. Formation of TiN: once the deposition temperature reaches 900 °C, N_2_ is introduced into the furnace and reacts with the Ti atoms to form TiN using a gas–solid reaction:
  $${\mathrm{TiO}}_{2}\overset{900\;{}^{\circ}C}{\underset{N2}{\to }}{\mathrm{TiN}+O}_{2}(\mathrm{outlet})$$
3. Adsorption of TiN on the substrate: once the reaction is over, the TiN molecules are transported to the surface of the Si substrate using diffusion and adsorbed onto the surface.
4. Nucleation and growth of TiN: The adsorbed TiN molecules act as nucleation sites for the growth of the TiN coating. As the deposition continues, the TiN coating grows and covers the entire surface of the Si substrate.

### 3.2. Characterisation Techniques

The elemental and morphological evaluation of TiN film deposited over Si substrate was accomplished using scanning electron microscope (SEM) model EVO MA18. The equipment was also attached using Oxford energy-dispersive X-ray spectroscopy (EDS) (X-act). The coating roughness and surface topography were evaluated using atomic force microscopy (AFM) with an INNOVA SPM instrument equipped using a 100 µm scanner. The film thickness was estimated using a Veeco Dektak-300 profilometer (Plainview, NY, USA) equipped with a 2.5 µm radius diamond stylus. While measuring thickness, the stylus was moved from one end to another end of the coating for five consecutive locations. The thickness shown for each sample was taken by considering the median step height. For evaluating the various nanomechanical properties such as H, E, plasticity index, etc., the well-known nanoindentation technique using nanoindenter model NHTX 55-0019 (Needham Heights, MA, USA) nanohardness tester provided with Berkovich diamond indenter tip (B-I 93; the radius of curvature 20 μm) was used. Special care was taken during the nanoindentation test regarding the indenter penetration over the sample, as during loading, the indenter was not allowed to penetrate beyond 10% of the coating thickness in any case. The maximum indentation load was kept at 3 mN. The overall deformation behaviour of each coating was evaluated using a loading–unloading rate of 0.67 mN/s, a 50 m ns^−1^ acquisition rate and nominal strain of 0.011 s^−1^ with a dwell time of 2 s.

## 4. Results and Discussion

### 4.1. Elemental Compositional Evaluation Using Energy-Dispersive Spectroscopy

**Figure 1:**
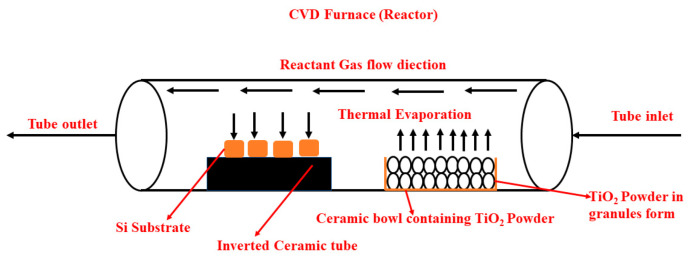
Schematic representation of CVD ceramic tube installed inside the CVD furnace.

**Figure 2:**
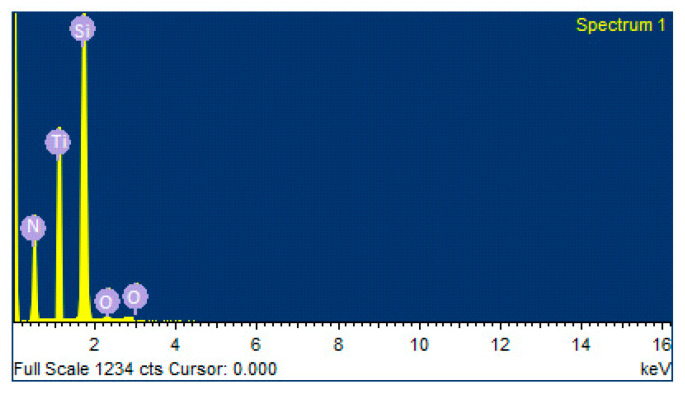
EDS results showing various elements in TiN coating.

Figure 1 and Table 2 show the elemental investigation of TiN coating. The presence of Si (being the parent substrate) was maximum, followed by Ti and N. There were also traces of oxygen observed. With the increase in deposition time, both Ti and O in TiN coating increased. In Figure 2, samples investigated using EDS at 200 nm revealed that the coating was mainly composed of titanium (Ti) and N_2_, with very few traces of oxygen. The presence of oxygen may be associated with Ti as well as TiO_2_.

### 4.2. Morphological Analysis Using Scanning Electron Microscopy (SEM)

**Figure 3:**
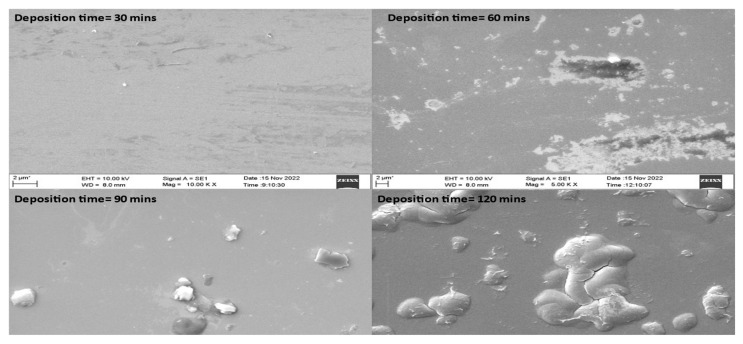
SEM images of TiN coatings grown over Si substrate at varying deposition times.

**Figure 4:**
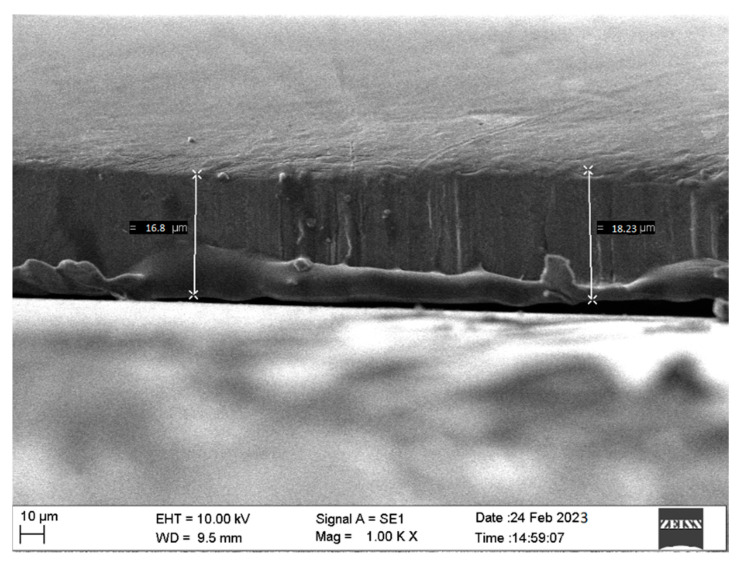
SEM cross-section image of TiN coating grown over Si substrate.

Figure 3 presents microstructural analyses of TiN coatings that were prepared using varying deposition times. SEM images of TiN coatings were recorded at 2 µm scales. Coatings prepared using varying deposition times showed a thick TiN layer over the substrate along with agglomeration [24]. The TiN coating that was prepared at 120 min deposition time exhibited smoother surface morphology compared to coatings prepared at 30, 60 and 90 min deposition time. It was also observed that with the increase in deposition time, the coating surface subsequently achieved higher roughness. The enhanced surface roughness with change in deposition time may be due to higher turbulence of the gaseous particles during the growth of the thin film. The TiN coating deposited using varying deposition times was also found to have agglomeration over the surface. Using 90 and 120 min deposition time, small particles in the form of agglomeration were found to be spread over the sample. However, the coating grown using 60 min deposition time also showed nonuniform deposition with small pits. For coatings grown at 30 and 60 min deposition time, a large number of pits along with agglomeration was observed. Figure 4 shows the SEM cross-section image of TiN coating grown over Si substrate.

### 4.3. Surface Roughness Analysis Using Atomic Force Microscopy (AFM)

**Figure 5:**
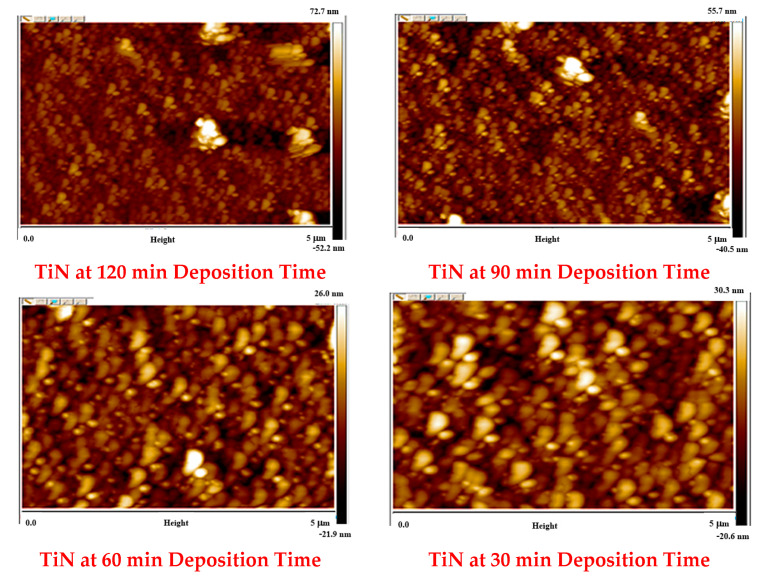
Two-dimensional images of TiN coatings grown over Si substrate at varying deposition times.

The AFM characterisation of TiN samples grown at varying deposition times is shown in Figure 5 and Table 3. The TiN sample images obtained are in good agreement with the TiN SEM images discussed in Section 3.2. AFM results also confirmed an increase in surface roughness of the TiN coating with increasing deposition time. Moreover, the sample images captured at 5-micron scale show the TiN particle size (maximum and average) synthesised at 30, 60, 90 and 120 min are 187.2 nm and 43.4 nm, 169.4 nm and 31.8 nm, 168.1 nm and 29.22 nm, and 159.44 nm and 29.32 nm, respectively. The reduced particle size with the increase in deposition time is directly interlinked with the higher duration of N_2_ low in the CVD chamber during deposition. The increase in deposition time also resulted in an enhancement in particle distribution density. Based on the information provided, the enhanced particle distribution density and presence of small pores or voids are contributing factors to the increase in surface roughness with increasing deposition time. This is because the increased density of particles in the coating, especially the tiny gaseous particles over the voids between the large particles, can create a more uneven surface with small pores and voids [25]. As a result, the surface roughness, as measured by Ra, increases. It is also possible that other factors, such as the deposition process itself or the characteristics of the TiN coating material, may also contribute to the observed increase in surface roughness.

### 4.4. Evaluation of Load versus Displacement Curve along with Nanomechanical Characteristics of TiN Coating Deposited Using Varying Deposition Time

**Figure 6:**
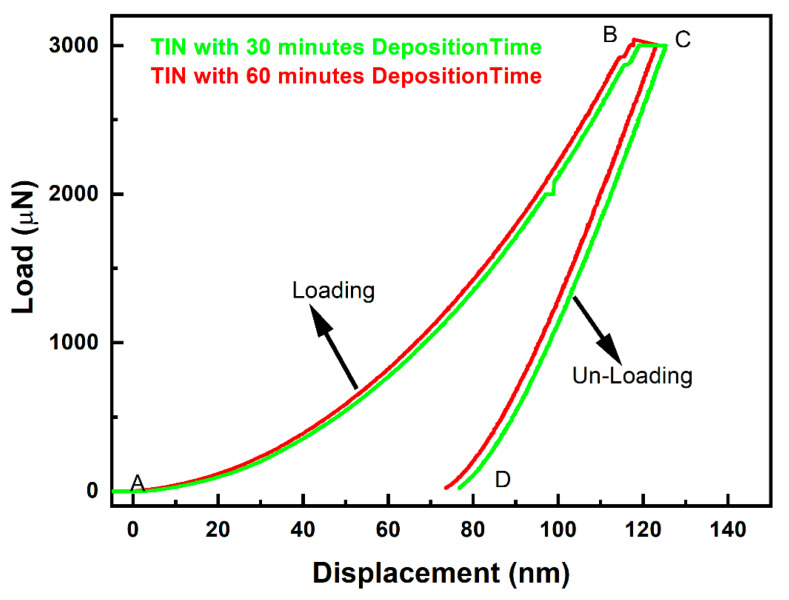
Load displacement curve for TiN deposited at 30 and 60 min deposition time.

In Figure 6 and Table 4, the load versus displacement curve of TiN coatings synthesised at 30 min and 60 min deposition time is shown. The curve rises from A to point B, indicating indenter loading onto the sample surface, from B to C indenting the sample at the same peak load for a duration of 10 s and then dropping from point C to point D, indicating the unloading of the indenter from the coating. The sample was tested using a maximum indentation load of 3000 µN or 3 mN. During the nanoindentation test, the maximum load was kept constant. During indenter penetration, the maximum depth was restricted below 10% to mitigate the influence of the substrate’s hardness. This was carried out with utmost care to ensure accurate results. The P–H curve in Figure 6 for TiN coating deposited at 30 and 60 min can be seen to have different displacement for the same indentation load. For the coating grown at 30 min deposition time, the maximum displacement in the P–H curve observed is 125.22 nm, whereas for the coating grown at 60 min deposition time, the maximum displacement in the P–H curve observed is 123.16 nm. This variation in displacement for the same load is due to the increase in enhancement in hardness of the coating with increasing deposition time.

The load versus displacement plot provided by the Oliver–Pharr approach [26].

(1) $$P=\alpha \;\left(h-hf\right)\;m,n$$

where P is indentation load; α is load–unload fitting parameter; h is indenter displacement; hf is the indenter parameter; m, n are the displacement exponent in the P–H relation for loading–unloading. A smooth P–H curve can be seen in Figure 6. However, a sudden pop-in P–H curve of TiN coating deposited at 30 min deposition time is observed. However, no such case is observed in case of the coating deposited with 60 min deposition time. The pop-in phenomena observed may be attributed to sudden and sharp displacement of the indenter into the sample. This phenomenon could have occurred due to localised region of the sample possibly having undergone a deformation process such as phase transformation or dislocation nucleation. This further led the coating to undergo rapid stress relaxation, which leads to the sudden displacement of the indenter.

The *H* and *E* value of TiN film deposited using varying deposition times was evaluated using the equation mentioned below:

(2) $$H=\frac{P_{max}}{A}$$

(3) $$S=\frac{dp}{dh}=2\beta \sqrt{\frac{A}{ℼ}}\;Er$$

(4) $$\frac{1}{E_{r}}=\frac{1-v_{f}^{2}}{E_{f}}=\frac{1-v_{id}^{2}}{E_{id}}$$

Here, $P_{max}$ is the maximum load over the sample provided by the indenter, *A* is area, in Equation (3), which is known as the Sneddon equation [27]. S is contact stiffness, β is the indenter constant and is considered as 1.034 for the Berkovich indenter, *Er*: elastic modulus and the subscript *id* and *f* mentioned in Equation (4) represent the indenter and film, respectively. The *H* and *E* values of the TiN coating are calculated by taking Young’s modulus and Poisson’s ratio of the indenter as 1141 GPa and 0.07 [28], respectively, and Poisson’s ratio value for TiCN thin film is considered as 0.30. With the increase in deposition time, it was observed that the *H* and *E* of the TiN thin film increases. This could be due to the higher volume deposition of Ti and N atoms with increasing deposition time. The results obtained in this case are in good agreement with the SEM images shown in Figure 3, where with increase in deposition time, the intensity of pores seen over the coating were reduced gradually, resulting in the formation of a smoother coating with higher deposition time. However, the increase in hardness of the coating may also depend on various other factors such as coating adhesion over the substrate and flow rate of the precursor gas.

**Figure 7:**
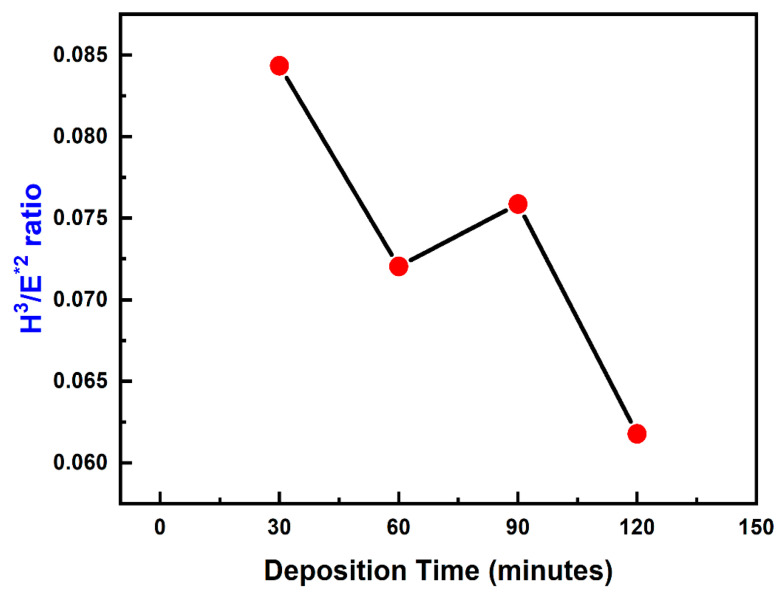
Plastic deformation resistance (H^3^/E^2^) of TiN coatings using varying deposition times.

Figure 7 shows the nature of TiN coatings in resistance to plastic deformation (H^3^/E^2^) with respect to varying deposition time. From the graph, it can be seen that with an increase in deposition time there is a consecutive reduction in H^3^/E^2^. It has been reported that the yield strength of the material is directly proportional to H^3^/E^2^ and therefore as the resistance to plastic deformation reduced, this also resulted in a relative decrement in yield strength of the coating.

### 4.5. Residual Stress of TiN Coating Deposited Using Varying Deposition Times

**Figure 8:**
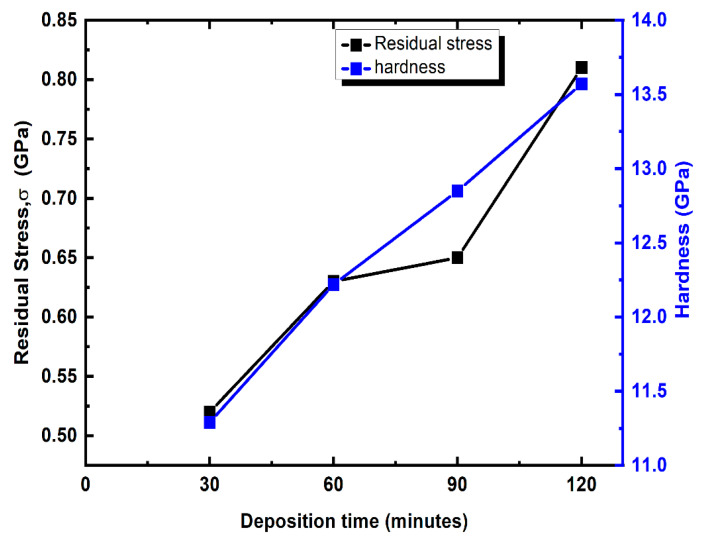
Hardness and internal stress variation in TiN coating with respect to deposition time.

Figure 8 illustrates the variation in hardness and internal stress (σ) of TiN coating at various depositing times. Here, the Stoney equation is used to calculate the σ of the TiN coating and is presented in Equation (5).

(5) $$\sigma =\frac{E_{S}}{6\left(1-{ʋ}_{s}\right)}\times \frac{t_{s}^{2}}{t_{nc}}\left(\frac{1}{R_{i}}-\frac{1}{R_{f}}\right)$$

where $t_{nc}$ is the thickness of the TiN coating and $t_{s}$ is the thickness of the Si substrate. $R_{i}$ is the curvature of the coating and $R_{f}$ is the curvature of the substrate. The Young’s modulus of the substrate is denoted as $E_{S}$ and the value is 127 GPa. The poisson ratio of the substarte is denoted as ${ʋ}_{s}$ and the value is 0.27 [45]. From Figure 8, it is observed that the residual stress of the coating increases with the increase in deposition time. The minimum and maximum σ are 0.52 GPa and 0.81 GPa for the TiN having deposited times of 30 and 120 min, respectively. The residual stress is compressive in nature and the value of σ increases due to the increase in Ti wt. % with respect to deposition time [14].

## 5. Conclusions

TiN thin film coatings were deposited over Si substrate using the CVD technique with variations in deposition time from 30 to 120 min. The SEM images indicated a relative decrement in pores/inclusions over the coating surface. Moreover, SEM images showed presence of agglomeration over the coating surface due to sudden melting and evaporation of powder particles over the sample substrate. The coating thickness was also found to be increased. The surface roughness of the coating evaluated using AFM was observed in the range of 21.26 to 29.68 nm. The mechanical properties evaluated using nanoindentation technique showed *H* and *E* of the coating in the range of 11.29 to 13.97 GPa and 130.63 to 201.13 GPa, respectively. An increase in deposition time also resulted in the consecutive reduction in yield strength of the coating. The residual stress of the developed composite increases due to the increase in Ti wt. % with respect to deposition time.

## Figures and Tables

**Table 1:** Critical Research articles related to TiN and CVD process along with main findings.

Authors	Deposition	Findings
Azadi et al. [41]	PACVD	Homogenous coating, pinhole-free coatings, fine-grained layer structure, higher hardness (27 GPa), wear resistance observed.
Krishna et al. [17]	PVD, PECVD	TiN/CNT hardness (3.18 GPa/2.8 GPa) thermal heat flux CNT/TiN 1570 W/mm^2^.
Cheng and Wen [11]	CVD	Higher deposition temperature resulted in twin crystal-free coatings, low porosity, smaller grain size, hardness 2000H_v_.
Das et al. [20]	CVD	Increase in N_2_ flow rate resulted increased surface roughness from 12.42 to 28.56 nm, TiN *H* and *E* observed as 30.14 GPa and 471.85 GPa.
Das et al. [26]	CVD	TiN hardness and Young’s modulus observed as 27.22 GPA and 355.11 GPa, respectively. Higher deposition temperature resulted in poor resistance to corrosion of TiN coating.
Cheng et al. [42]	CVD	Lower deposition temperature resulted in the formation of twinned crystals. Higher deposition temperature resulted twin free crystals.
Baltatu et al. [43]	Biomimetic	Deposited hydroxyapatite coating over titanium alloys. Results revealed enhancement in osteointegration materials used as implants.
Pintelia et al. [44]	Atmospheric plasma spraying	Al_2_O_3_ with 99.5% purity and ZrO_2_/20%Y_2_O_3_ were deposited over AA2024 aluminium alloy. The coated ZrO_2_/20%Y_2_O_3_ sample showed ductile fracture behaviour and Al_2_O_3_ showed brittle fracture behaviour.

**Table 2:** TiN coating elemental analysis grown using varying deposition times.

Sample Compositions	TiN 30 min	TiN 60 min	TiN 90 min	TiN 120 min
Si (at. %)	49.26	50.24	49.95	49.16
Ti (at. %)	24.15 ± 2.21	25.22 ±1.88	25.98 ± 0.89	26.12 ± 0.25
N (at. %)	9.63 ± 1.67	10.32 ± 1.17	10.98 ± 1.12	11.31 ± 0.76
O (at. %)	16.96 ± 1.08	14.22 ± 0.81	13.09 ± 0.56	13.41 ± 0.42

**Table 3:** TiN sample surface roughness synthesised at varying deposition times.

Sample Code Roughness (nm)	TiN at 30 min Deposition Time	TiN at 60 min Deposition Time	TiN at 90 min Deposition Time	TiN at 120 min Deposition Time
R_a_	21.26 ± 0.54	25.12 ± 0.23	29.28 ± 0.63	29.28 ± 0.63
R_z_	31.81 ± 1.95	49.13 ± 2.63	52.52 ± 2.95	52.96 ± 3.18
R_q_	23.82 ± 2.11	25.42 ± 1.56	32.74 ± 2.28	33.36 ± 2.74

**Table 4:** Hardness and Young’s modulus values of TiN coatings.

Deposition Time	Hardness	Young’s Modulus
TiN at 30 min deposition time	11.29 ± 0.56	130.63 ± 6.53
TiN at 60 min deposition time	12.22 ± 0.61	159.17 ± 7.95
TiN at 90 min deposition time	12.85 ± 0.642	167.25 ± 8.36
TiN at 120 min deposition time	13.57	201.13

## Data Availability

The data presented in this study are available in the article.
